# Improving the scalability of psychological treatments in developing countries: An evaluation of peer-led therapy quality assessment in Goa, India

**DOI:** 10.1016/j.brat.2014.06.006

**Published:** 2014-09

**Authors:** Daisy R. Singla, Benedict Weobong, Abhijit Nadkarni, Neerja Chowdhary, Sachin Shinde, Arpita Anand, Christopher G. Fairburn, Sona Dimijdan, Richard Velleman, Helen Weiss, Vikram Patel

**Affiliations:** a 1205 Ave Docteur Penfield, Department of Psychology, McGill University, Montreal, Quebec, H3A 1B1, Canada; b Sangath, H No 451 (168), Survey No 50/31, Succour, Porvorim, Bardez, Goa 403501, India; c Centre for Global Mental Health, London School of Hygiene & Tropical Medicine, Keppel Street, London, WC1E7HT, United Kingdom; d Department of Psychiatry, Warneford Hospital, Oxford, OX3 7JX, United Kingdom; e University of Colorado, Boulder, 345 UCB Muenzinger, Boulder, CO 80309-0345, USA; f Department of Psychology, 2 South, University of Bath, Bath, BA2 7AY, United Kingdom; g Centre for Chronic Conditions and Injuries, Public Health Foundation of India, New Delhi, India

**Keywords:** Therapy quality, Lay therapists, Peers, Supervision, Competency

## Abstract

Psychological treatments delivered by lay therapists, with little or no previous mental health training, have been shown to be effective in treating a range of mental health problems. In low resource settings, the dearth of available experts to assess therapy quality potentially leads to a bottleneck in scaling up lay therapist delivered psychological treatments. Peer-led supervision and the assessment of therapy quality may be one solution to address this barrier. The purpose of this study was two-fold: 1) to assess lay therapist quality ratings compared to expert supervisors in a multisite study where lay therapists delivered two locally developed, psychological treatments for harmful and dependent drinking and severe depression; 2) assess the acceptability and feasibility of peer-led supervision compared to expert-led supervision. We developed two scales, one for each treatment, to compare lay therapist and expert ratings on audio-taped treatment sessions (*n* = 189). Our findings confirmed our primary hypothesis of increased levels of agreement between peer and expert ratings over three consecutive time periods as demonstrated by a decrease in the differences in mean therapy quality rating scores. This study highlights that lay therapists can be trained to effectively assess each other's therapy sessions as well as experts, and that peer-led supervision is acceptable for lay therapists, thus, enhancing the scalability of psychological treatments in low-resource settings.

## Highlights

• Peer lay therapists can assess therapy quality as well as experts.
• Peer-led supervision is preferred to expert-led supervision by lay counsellors.
• Scalability of psychological treatments in low-resource settings can be enhanced by using peer-led supervision.

## Introduction

Psychological treatments delivered by lay therapists, with little or no previous mental health training or experience, have been shown to be effective in addressing a range of mental health problems in low and middle income countries (van Ginneken et al., 2013). Successful interventions can be based on empirically-supported cognitive, behavioural and interpersonal techniques that are adapted for the local context and involved well-defined supervision protocols led by experts (Patel, Chowdhary, Rahman, & Verdeli, 2011). Supervision is considered a key, pedagogical and quality assurance tool in treatment delivery (Bernard & Goodyear, 2009; Schoenwald et al., 2013, Waltz et al., 1993). Experts, typically mental health professionals who are experienced and trained in specific treatment modalities, are generally recognized as the gold standard in assessing supervisees' ability (whether lay supervisees or more junior mental health professionals) to deliver psychological treatments with acceptable quality (Townend, Iannetta, & Freeston, 2002). During or after an individual session, expert supervisors may provide supervision to lay therapists, with performance feedback and coaching, to maintain and further develop their skills (Baer et al., 2007). Multiple models for supervision exist and can vary across a number of variables including format (e.g., group vs. individual) and frequency (weekly vs. monthly).

Despite these advantages, experts in psychological treatments are not readily available (Kilminster & Jolly, 2000), particularly in developing countries (Patel et al., 2010). One alternative to expert supervision is self-assessment. Self-assessment requires fewer resources in terms of time and availability and may assist lay therapists to learn new skills by monitoring their own performance (Muse & McManus, 2013); however, accuracy of self-assessment has been questioned, with evidence suggesting a tendency among therapists in training to either overestimate (e.g., Brosan et al., 2008, Perpletchikova and Kazdin, 2005) or underestimate their therapy quality (e.g., McManus, Rakovshik, Kennerley, Fennell, & Westbrook, 2012).

One solution to the dearth of experts and questionable accuracy of self-assessment may be peer-led supervision. While several models and definitions exist (see Borders, 2012), peer-led supervision has been argued to be advantageous because it encourages therapists to draw upon others' experiences and take active roles in assisting one another including the alleviation of stress, anxiety and feelings of inadequacy (Yeh et al., 2008). Although there is enthusiastic support for peer models (e.g., Golia & McGovern, 2013), most research involving peer-led supervision have not been empirically tested against other supervision models in psychotherapeutic settings (Borders, 2012, Newmann et al., 2013). However, initiatives in higher education, such as the online learning platform Coursera, have demonstrated high levels of agreement between the marks given by peers (equivalent to our ratings of quality) and those of experts (https://www.coursera.org/).

The extent to which lay therapists, experts, and their peers agree about the quality of individual therapy sessions could inform practice guidelines to successfully scale up psychological treatments. For example, if peers of lay therapists could be trained to reliably evaluate sessions similarly to experts, then peer-led supervision may be the most cost-effective approach for assessing therapy quality. In the current study, we use the term “peers” to refer to the peers of lay therapists.

The objectives of the current study were two-fold: 1) to examine the agreement between expert, self, and peer therapy quality ratings of individual treatment sessions for harmful and dependent drinking and for severe depressive disorders, delivered by lay therapists in primary care in Goa, India; and 2) evaluate the acceptability of peer-led compared to expert-led supervision. Specifically, the study aimed to: a) describe the development, inter-rater reliability and internal consistency of therapy quality scales for each treatment; b) to estimate the agreement of peer and self-ratings of therapy quality against those of experts; and c) assess lay therapists' perspectives of peer-led compared to expert-led supervision across three time periods (stages) over ten months. We hypothesized that, with increasing therapist competency, the differences between lay therapist and expert ratings of therapy quality would reduce significantly.

## Methods

### Setting

This study was conducted in 11 purposively selected primary health centres (PHC) in Goa, India. The study is part of PREMIUM (PRogramme for Effective Mental health Interventions in Under-resourced health systeMs) which aims to develop and evaluate the effectiveness of two brief, contextually-appropriate psychological treatments for harmful and dependent drinking (Counselling for Alcohol Programme (CAP)) and depressive disorders (Healthy Activity Programme (HAP)) delivered by lay therapists (see Patel et al., 2014). Manuals for both treatments are available online (http://rubiqhosting.com/sangath/images/manuals/). Ethical approval for PREMIUM was granted by the Institutional Review Boards of Sangath and the London School of Hygiene and Tropical Medicine, and the Indian Council of Medical Research.

### Lay therapists

**Fig. 1:**
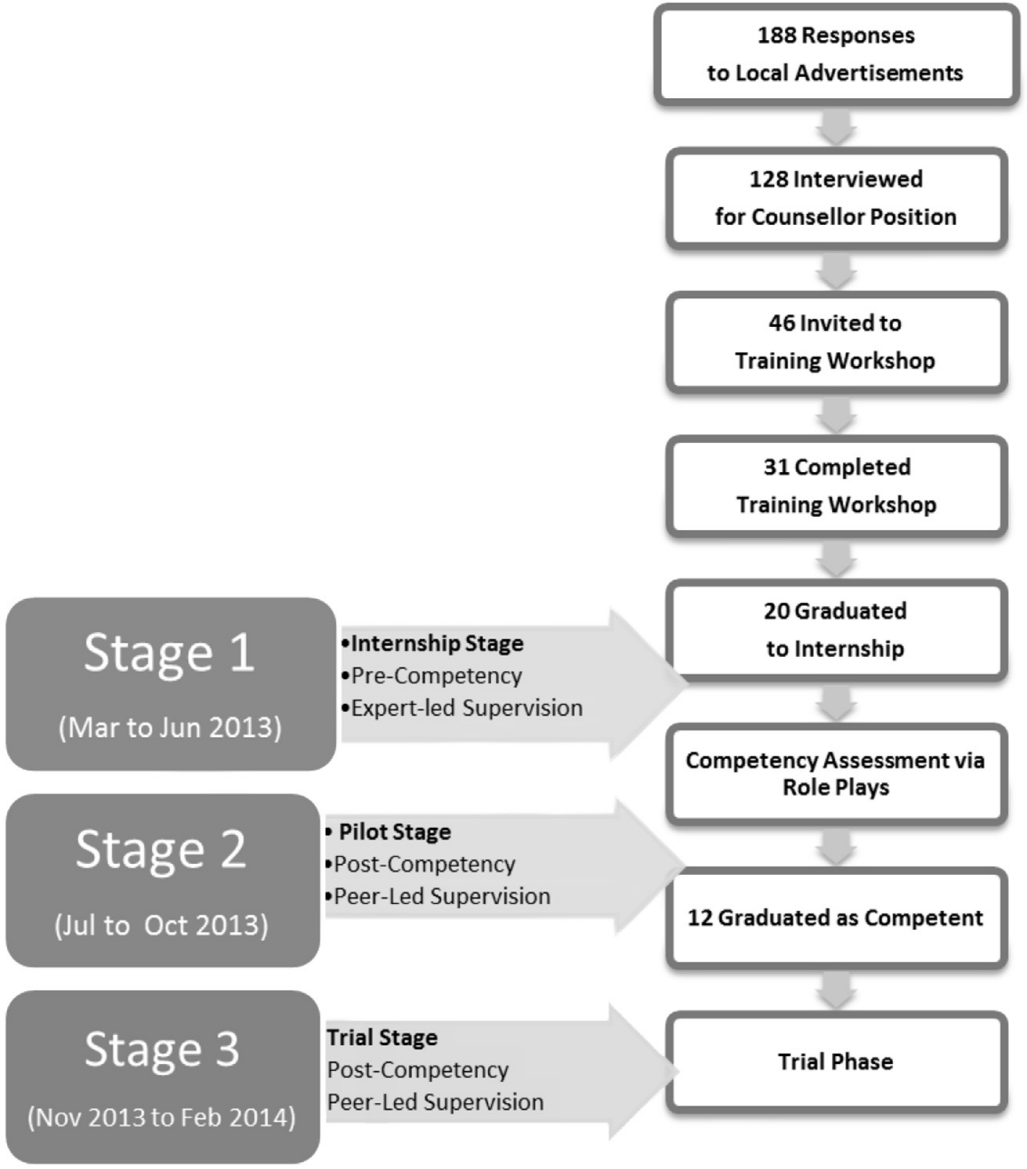
Lay therapist recruitment, training and study stages.

The selection of lay therapists is outlined in Fig. 1. Lay therapists were recruited through advertisements in newspapers and a local television channel. A total of 188 applicants responded and 128 prospective candidates were selected by mental health experts to be interviewed. Exclusion criteria were any formal training or qualification in a health profession. Essential criteria were the completion of tenth grade education and fluency in local languages. Desirable criteria were having a higher education beyond tenth grade, lack of prior professional training in mental health, and a two-year commitment to the pilot and future trial. The interview entailed a structured questionnaire and a brief role play in which candidates were asked to counsel a friend. Lay therapists were evaluated based on their willingness to be part of a team, communication and interpersonal skills. Following the interview, 31 candidates were invited and completed the training.

Training comprised a three-week workshop focused on three domains: general counselling skills and the two manualized treatments (http://www.sangath.com/images/manuals). Training involved lectures, demonstrations by trainers, and practice of specific skills via role plays. Lay therapists' knowledge was assessed via a multiple choice exam (cut-off scores = 80% on 150 questions) as well as their performance on role plays using standardized vignettes. Of the 31 lay therapists who completed the training, 20 were selected for the Internship Stage (see below and Fig. 1). One person dropped out shortly before the Internship Stage which therefore began with a total of 19 lay therapists.

On average, lay therapists were 25.9 years of age with 15 years of education; sixteen were female. The Internship Stage (March to June 2013) involved the implementation of HAP and CAP treatments in the PHC setting with the lay therapists in training being supervised in groups by experts. At the end of the Internship Stage, twelve trainees, who achieved competence viz. standardized role plays, were selected for the Pilot Stage (July to October 2013). The Pilot Stage involved the continued treatment of patients in the PHC setting with one significant modification: group supervision was now led by a peer rather than an expert. The Pilot Stage was followed by the Trial Stage which began on October 28th, 2013 and will continue until July 2015. The current study therefore consists of data rating individual treatment sessions, from a total of19 lay therapists, from three progressive stages: the Internship Stage (Stage 1); the Pilot Stage (Stage 2); and the initial period (Nov 2013 to Feb 2014) of the Trial Stage (Stage 3).

### Outcome measures of therapy quality

Our definition of therapy quality is based on that by Fairburn and Cooper (2011), who define therapy quality by whether a psychological treatment is delivered well enough for it to achieve its specific effects. In the current study, this means whether lay therapists can be trained to evaluate individual treatment sessions as well as experts. Furthermore, therapy quality refers to not only whether a lay therapist has implemented the appropriate treatment, but also whether they have done the ‘right things well’ (p. 379).

One way to assess therapy quality is to ask lay therapists to rate an individual session using tools that measure *treatment-specific skills* – elements prescribed for a specific therapeutic modality – and *common skills* – elements shared across therapeutic modalities (Fairburn and Cooper, 2011, Webb et al., 2010). Treatment-specific factors are essential to the implementation of a particular treatment modality and can be assessed for a particular phase of treatment (i.e., beginning, middle or end phases). Common skills include factors such as engaging clients as well as forming and maintaining a good therapeutic alliance, irrespective of what disorder and treatment modality are being used (Webb et al., 2010). Steps for scale development are outlined below.

The development of both therapy quality measures were originally based on existing scales reflecting core treatment components of the two manualized treatments. Using a literature search, we first aimed to identify the most applicable scales to assess Motivational Interviewing (MI) for the Counselling for Alcohol Programme (CAP) and Behavioural Activation (BA) for Healthy Activity Programme (HAP). For CAP, we selected the Motivational Interviewing Target Scheme 2.0 (MITS; Allison, Bes, & Rose, 2012), to measure MI techniques. MITS measures 10 practitioner behaviours that amount to a comprehensive description of observable practice of MI-specific and general techniques (e.g., activity emphasis, posture, empathy, collaboration, evocation, independence, navigation, and information/advice). The Quality of Behavioural Activation Scale (Q-BAS; Dimidjian, Hubley, Martell, Herman, & Dobson, 2012) selected for HAP is a scale to measure competence and is modelled after the Cognitive Therapy Rating Scale (Young & Beck, 1980). The Q-BAS assesses the quality of how well BA-specific and general skills are implemented in 14 domains including (1) Structural and Stylistic Strategies (e.g., establishes and follows agenda, nurtures activation, learns together as a team, is non-judgemental and matter of fact); and (2) Conceptualization, Strategy and Application (e.g., uses the BA model throughout treatment, reviews homework selects promising targets for change, and applies strategies skillfully).

We then consulted international experts (RV and SD) to determine how these scales could be best appropriated for the two PREMIUM treatments. The revised scales were then piloted to local experts and international experts who co-rated similar sessions until there was a high level of agreement on scale items. Further modifications of the scales were carried out to enhance reliability and utility including a simplification of terms and the integration of contextually derived CAP and HAP treatment areas (e.g., the inclusion of cognitive and behavioural therapeutic practices in the CAP and the engagement of significant others).

Eventually, the Quality of the Counselling for Alcohol Programme (Q-CAP) scale and the Quality of the Healthy Activity Programme (Q-HAP) scale were finalized as our two therapy quality measures. In both measures, two subscales were used to assess overall therapy quality. Treatment-Specific Skills (TSS), including phase specific skills, were specific to the HAP or CAP treatments; whereas General Skills (GS) assessed the common skills used in either treatment and therefore the identical subscale was used in both disorder scales (further described below). Each item is measured on a 5-point Likert scheme (0 *not done* to 4 *excellent*) with the rating scheme of 0–4 that was operationalized to enhance reliability. “*Not applicable*” served as another option for lay therapists as certain items, for example the involvement of significant others, may not have been rated as applicable to all sessions.

The Q-CAP and Q-HAP scales were then piloted with 19 lay therapists who entered the first phase of the current study (see below). They provided feedback about the layout, content and utility of specific scale items. Furthermore, lay therapists were trained to use both measures, during which they were explained the meaning of each item with specific examples, and had an opportunity to test the scales by assessing individual treatment sessions during group supervision. Feedback was provided to the research team and further modifications were made related to the utility of the scales (e.g., wording and numbering of specific questions). Complementary to the definition of therapy quality above, analyses of treatment-specific scales included phase-specific items. This was confirmed using an analysis of Cronbach's alpha (*α*) which resulted in a poor internal consistency for phase-specific only items on both the Q-CAP (*α* = .51) and Q-HAP (*α* = .52); however, when combined with treatment-specific items, internal consistency of the treatment-specific subscale improved from *α* = .72 to *α* = .87 on the Q-CAP and *α* = .75 to *α* = .89 on the Q-HAP.

*The General Skills subscale* which was common to both scales was adapted from common items measuring the lay therapist's approach across both Q-BAS and MITS 2.0, the Counselling Skills Scale (CCS; Dimick & Krause, 1998) and specific to the Counselling Relationship Manual (http://www.sangath.com/images/manuals/Counselling%20Relationship_Manual.pdf). Modifications from the original scales were implemented to enhance the practical utility of the scale, remove items that were not consistent across the Q-HAP and Q-CAP scales and to reduce any redundancy across all the items. *The General Skills subscale* comprises 10 items related to the lay therapist's general approach to therapy. For example, lay therapists were assessed on the extent to which they expressed empathy, used a collaborative style, expressed a non-judgemental attitude and used open-ended questions.

*The CAP Treatment-Specific subscale* comprises 22 items assessing a range of CAP treatment skills, for example, assisting patients to develop a change plan, working on drinking refusal skills and handling the urge to drink.

*The HAP Treatment-Specific subscale* comprises 15 items assessing a range of HAP treatment skills, for example, whether and how well the lay therapist explained the HAP model to the patient and conducted mood monitoring in a given treatment session.

The Q-CAP and Q-HAP can be found at http://www.sangath.com/images/manuals/Q%20HAP.pdf and http://www.sangath.com/images/manuals/QCAP.pdf respectively.

### Sample size calculation

A sample size of 30 individual treatment sessions per stage would provide 80% power to detect a mean difference of .3 points (based on the full 5-point range of 0–4 in our therapy quality scales, this represents a proportionate difference of about 5%), with a SD of .6.

### Procedure

#### Assessment of therapy quality

Individual treatment sessions were audio recorded with the patient's consent. On average, each audio session lasted 43.0 min (95% CI: 40.8–45.2). Approximately 2% of therapy quality sessions were rejected due to poor sound quality. Because this assessment of therapy quality played a key role in training lay therapists, individual treatment sessions were purposively selected by experts to ensure equal distribution across lay therapists and treatment phases. Supervision was implemented on a weekly basis. Typically, groups consisted of one expert, three to four peers and the lay therapist who conducted the session. During any given supervision session, lay therapists were divided into similarly sized groups which lasted up to a maximum of 90 min including feedback and discussion. In Internship Stage 1, one to two experts moderated the supervision and therapy quality assessments in each group; in Pilot and Trial Stages 2 and 3, one peer (chosen in rotation) performed the same tasks. In both expert- and peer-moderated supervision of lay therapists, one individual audio-recorded session was listened to in full and then rated, using the scales described above, independently by experts and by each peer. Self-ratings were completed prior to group supervision to reduce bias as a consequence of the supervision. Once the session was listened to and rated, ratings were discussed and feedback was provided by all group members to the lay therapist whose tape was being rated. Notably, while the same lay therapists delivered the two treatments and participated in the supervision sessions, there were a different group of experts for each of the two treatments.

There were six experts in total including two psychiatrists, two clinical psychologists and two senior therapists who had previously provided psychological treatment as part of the MANAS trial (Patel et al., 2010). Experts had an average of 9.75 and 5.42 years of experience delivering and supervising psychological treatments, respectively. Experts were clinicians who are well-versed in manualized, evidenced-based treatments used in developing country settings including cognitive, behavioural and interpersonal techniques. Prior to training the lay therapists for the current study, experts were trained in person in the core psychological treatments (Behavioural Activation for depression and Motivational Interviewing for harmful drinking) by international experts and continued to receive monthly supervision from them in person or via Skype. Experts were found to be competent in the delivery of these treatments by international experts.

#### Assessment of peer-led supervision

Focus group discussions (FGDs) were conducted with lay therapists to determine their perspectives on peer-led supervision in comparison to expert-led supervision. In total, three FGDs were conducted, two in the Internship Stage 1 and one at the end of Pilot Stage 2. Using a guided semi-structured interview, an experienced qualitative researcher (SS) asked the lay therapists involved in the current study about their experiences of the two types of supervision formats, the challenges and difficulties and how these could be addressed. All three FGDs involved interviewing 19 lay therapists in one group simultaneously and the duration of each FGD lasted, on average, 75 min.

### Analysis

Three subscale scores were computed for the analyses in this paper: the General Skills Scale (GS) where observations from both the Q-HAP and Q-CAP are considered together; and the individual Treatment Specific scales of the Q-HAP (Q-HAP TSS) and Q-CAP (Q-CAP TSS). Scale scores were generated as follows: the total of all item scores were divided by the number of items which had been scored to arrive at a mean scale score (which ranged from 0 to 4) and took into account “Not Applicable” as a potential option. This procedure allowed us to generate a comparable score for all raters, addressing the potential limitation of missing values because, for example, certain treatment-specific items may not have been applicable to particular sessions. We estimated inter-rater reliability and internal consistency of our scales following the method of Carroll et al. (1994).

Specifically, we estimated the internal consistency using the ratings of the experts across stages as well as from lay therapists in Stages 2 and 3 (as the lay therapists had achieved a priori competency standards by this time). We estimated intraclass coefficients (ICCs) to assess inter-rater reliability between expert and lay therapist peer and self-ratings. We then conducted paired *t*-tests to assess differences between raters' mean subscale scores. All analyses were carried out separately by Stage to determine whether agreement between the expert and peer ratings changed over time, in particular to test our a priori hypothesis. SAS 9.3 was used to conduct all quantitative analyses. Thematic analysis was used to analyse FGD data to inductively code categories related to the acceptability of peer-led group supervision in comparison to expert-led supervision.

## Results

In total, *n* = 189 audio sessions across both treatments from a total of 19 lay therapists were used for this study. On average, each lay therapist had 3.79 of their individual treatment sessions rated in Stage 1, 4.07 in Stage 2 and 5.45 in Stage 3 (the higher average per stage reflecting the smaller number of lay therapists graduating from one stage to the next). Sessions were evenly distributed across lay therapists and treatment phases. Per treatment modality, raters assessed 92 individual CAP sessions and 97 individual HAP sessions; 34, 27 and 31 sessions were rated by the Q-CAP and 38, 30, and 29 sessions were rated by the Q-HAP in Stages 1, 2, and 3 respectively.

### Internal consistency and inter-rater reliability

Cronbach's alpha (*α*) demonstrated high internal consistency between treatment-specific and general skills subscale items for both the Q-CAP and Q-HAP. Using expert ratings, high internal consistency between items resulted for the Q-CAP TSS (*α* = .867, *N* = 90), Q-HAP TSS (*α* = .886, *N* = 97), and GS (*α* = .896, *N* = 187). Similarly, we found high internal consistency of all subscales among peer ratings: Q-HAP TSS (*α* = .807, *N* = 97), Q-CAP TSS (*α* = .858, *N* = 92) and GS (*α* = .828, *N* = 189). Inter-rater reliability, based on random mean pair comparisons of peer ratings for the three sub-scales, showed moderate values for ICC estimates for all three subscales: Q-CAP TSS (ICC(2,3) = .608, *N* = 90), Q-HAP TSS (ICC(2,3) = .616, *N* = 97), and GS (ICC(2,3) = .622, *N* = 189). Two blinded ratings on the same individual treatment sessions were obtained for some individual HAP sessions, showing moderate agreement between experts: ICC(2,3) = .603, *N* = 44 for TSS and ICC(2,3) = .637, *N* = 44 on GS.

### Agreement between expert, self and peer lay therapist ratings

Mean treatment-specific and general skills scores as evaluated by expert, self and peer therapist ratings are presented in Table 1. Among peer and expert raters, mean therapy quality scores and the upper limit of their range improved across consecutive stages for all the subscales, and improved or remained constant for self-ratings.

**Table 1:** Expert, self and peer ratings of treatment-specific and general skills per stage.

Q-CAP treatment-specific skills						
Rater	Stage 1(*n* = 34)		Stage 2(*n* = 27)		Stage 3(*n* = 31)	
	Mean	Range	Mean	Range	Mean	Range
Expert	1.72 (.48)	.71–2.60	2.17 (.42)	1.15–3.11	2.18 (.57)	1.31–3.25
Self	2.02 (.56)	.86–3.08	2.36 (.52)	1.08–3.15	2.48 (.40)	1.77–3.22
Peer	1.88 (.43)	.92–2.76	2.24 (.33)	1.49–2.97	2.27 (.38)	1.50–3.11

Q–HAP treatment–specific skills						
Rater	Stage 1(*n* = 38)		Stage 2(*n* = 30)		Stage 3(*n* = 29)	
	Mean	Range	Mean	Range	Mean	Range
Expert	1.76 (.66)	.50–3.29	1.91 (.61)	.64–3.00	2.21 (.54)	1.33–3.10
Self	2.08 (.62)	.70–3.13	2.41 (.47)	1.13–3.14	2.40 (.39)	1.56–3.20
Peer	2.00 (.52)	1.13–3.53	2.20 (.43)	1.13–3.00	2.32 (.37)	1.57–3.12

General Skills (Q–HAP + Q–CAP)						
Rater	Stage 1(*n* = 72)		Stage 2(*n* = 57)		Stage 3(*n* = 60)	
	Mean	Range	Mean	Range	Mean	Range
Expert	2.16 (.59)	.70–3.40	2.30 (.46)	1.30–3.10	2.56 (.50)	1.60–4.00
Self	2.40 (.53)	1.20–3.40	2.56 (.43)	1.44–3.20	2.67 (.37)	1.80–3.40
Peer	2.36 (.36)	1.48–3.27	2.56 (.34)	1.65–3.20	2.67 (.24)	2.08–3.40

There was some evidence of a difference in the mean therapy quality score between experts and both self and peers, especially in Stage 1 and 2, for both the TSS and GS scores. By Stage 3, however, there was no significant difference in the mean scores on any subscale (Table 2). Specifically, across Q-CAP and Q-HAP treatment-specific and general skills scores, any statistically significant differences between expert-peer ratings in Stages 1 and 2 were reduced to non-significant differences by Stage 3. By Stage 3, statistically differences were found between expert-self ratings in only the Q-CAP treatment-specific subscale.

**Table 2:** Mean Differences of between Expert, Self and Peer Ratings per Treatment and Stage (*t* value, *p*).

Q-CAP treatment-specific skills						
Rater	Stage 1(*n* = 34)		Stage 2(*n* = 27)		Stage 3(*n* = 31)	
	Mean difference	*t(p)*	Mean difference	t*(p)*	Mean difference	*T(p)*
Expert vs. Self	−.31	−3.69***	−.25	−2.65**	−.30	−2.71*
Expert vs. Peers	−.16	−2.56*	−.11	−1.80	−.08	−1.12
Self vs. Peers	.15	1.68	−.12	1.63	.22	2.83**

Q–HAP treatment–specific skills						
Rater	Stage 1(*n* = 38)		Stage 2(*n* = 30)		Stage 3(*n* = 29)	
	Mean difference	*t(p)*	Mean difference	*t(p)*	Mean difference	*t(p)*
Expert vs. Self	−.33	−3.72***	−.49	.5.26***	-.20	−1.59
Expert vs. Peers	−.24	−3.43**	−.30	−4.93***	−.12	−1.26
Self vs. Peers	.08	.98	.21	3.10**	.08	1.10

General Skills (Q–HAP + Q–CAP)						
Rater	Stage 1(*n* = 72)		Stage 2(*n* = 57)		Stage 3(*n* = 60)	
	Mean difference	*t(p)*	Mean difference	*t(p)*	Mean difference	*t(p)*
Expert vs. Self	−.25	−3.79***	−.28	−3.74***	−.10	−1.42
Expert vs. Peers	−.21	−3.73***	−.09	−4.80***	−.10	−1.79
Self vs. Peers	.04	.67	.00	.11	.00	.12

Note. *p*-values are reported as follows. *p < .05, **p < .01 ***p < .001.

### Acceptability of supervision

In expert-led supervision, thematic analyses demonstrated some positive feedback from lay therapists including ‘*learning from others*’ and described supervisors as ‘*supportive’* in helping lay therapists to ‘*understand their role as lay therapists*’. They described supervision as a constructive learning environment as supervisors “*encouraged all questions and a lot of learning happened”*. Lay therapists also expressed multiple challenges including the perception of ‘*too much criticism*’ and ‘*feeling underappreciated*’ by expert supervisors in Internship Stage 1. Lay therapists reported a preference for smaller groups (three to four individuals) rather than larger groups (six to 10 individuals). In contrast, FGDs assessing lay therapists' perceptions of peer-led supervision identified many positive themes including bolstering self-esteem (e.g., “*helping to build confidence levels among lay therapists*”); a positive learning environment (e.g., “*forces us to use the mind*”); an emphasis on equality (“*sense of equality and responsibility*”) and a participatory learning environment (“*lots of learning as everyone participates*”). While some lay therapists reported feelings of anxiety about the anticipation of moderating supervision sessions, they expressed that they better understood ‘*the responsibility of being an expert*’ implying that lay therapists acknowledged and could now empathize with the challenges that experts experience in moderating supervision sessions.

## Discussion

Our study describes the first systematic attempt to evaluate the acceptability and concordance of peer-led supervision and therapy quality compared with expert-led supervision. The study employed two therapy quality scales purposively designed for the two contextually appropriate treatments for harmful drinking and severe depression. Both measures demonstrated robust reliability properties.

Our findings confirmed our primary hypothesis of increased levels of agreement between peer and expert ratings as demonstrated by a decrease in the differences in mean therapy quality scores between raters to non-statistical differences. This finding was common to both treatments, illustrating consistency of lay therapists' performance in both treatments. As expected, average therapy quality ratings did not reach maximum scores; however as noted above, we observed that the upper limit of the range of scores exceeded 3 (on a 4 point scale) by Stage 3 across lay therapists and treatments.

We found no empirical studies comparing peer and expert therapy quality ratings in psychotherapy milieus in either developed or developing countries. Nor did we find any studies developing or evaluating a process where lay therapists were trained to provide peer supervision to one another. However, relevant meta-analyses have been conducted to compare expert-peer, expert-self, and self-peer ratings of job performance in organizational settings (see Conway and Huffcutt, 1997, Harris and Schaubroeck, 1988). Consistent with Harris & Schaubroeck, we found a good agreement between expert and peer ratings which were higher than expert-self agreement. However, inconsistent with the results of this meta-analysis, we saw good agreement between self and peer ratings which were not significantly different. This finding is important because it demonstrates that while mean self-ratings were sometimes higher than peer and experts ratings, these differences were not statistically significant from peer ratings; thus, suggesting that self-ratings are not necessarily biased as suggested by other studies (e.g., Brosan et al., 2008). Further, similar to McManus et al. (2012) who assessed the relationship between self and expert ratings on individual cognitive behavioural therapy sessions, we also found a high level of agreement between self and expert ratings on two out of three therapy quality subscales. Therefore, our study supports the use of peer ratings and possibly self-ratings to assess therapy quality once peers are trained systematically.

Equally important, our qualitative study revealed that lay therapists expressed more positive perspectives towards peer-led supervision as compared to expert-led supervision. Coupled with the quantitative outcomes mentioned above, this finding confirms previous studies' support for peer-led supervision (e.g., Golia & McGovern, 2013; Yeh et al., 2008). The current study highlights lay therapists’ preference for a participatory environment emphasizing equality and learning from other lay therapists in peer-led supervision.

The limitations of the current study include its convenience sample, as individual treatment sessions were selected on the basis of audiotape quality as well as to ensure equitable distribution across lay therapists and treatment sessions. Thus, while our study lacks a random selection of therapy sessions, we did meet our goal to provide fair distribution of training across lay therapists, treatment phases and treatments. Another potential limitation is that raters (both lay therapist and experts) were not blind to the identity of the lay therapist whose treatment session was being rated. Third, there might have been a range restriction of ratings due to a “safety bias” by lay therapists and experts; however, this is unlikely given the wide and increased upper limit of scores between stages. Finally, while we have reported psychometric properties of reliability, we acknowledge that that our full scales have not been validated and we have not yet been able to conduct some psychometric tests. For example, we were unable to calculate interrater reliability scores for the Q-CAP ratings because there was only one expert per group rating a given session and resource limitations have meant that we have not undertaken test-retest analyses in the current study. These are practical limitations in developing country contexts, particularly when mental health professionals serving as experts are minimal. Regarding validity, however, because scales were derived from instruments which are used by other psychological treatment researchers worldwide, we have assumed that they possess a degree of validity by extension. In addition, we will use the methods proposed by Carroll and colleagues who assessed the validity of their scales using trial data after the PREMIUM trials are unblinded in late 2015 (Patel et al., 2014).

In conclusion, our study demonstrates that lay therapists can be trained to achieve concordance with experts in the assessment of individual therapy session quality, especially if they are rating the sessions of other lay therapists (as peers). We also showed that peer-led supervision can be implemented systematically in a format that is acceptable to and preferred by lay therapists. This study therefore highlights that lay therapists can be trained to effectively assess each other's therapy sessions as well as experts, enhancing the scalability of psychological treatments in low-resource settings.
